# Shock index and modified shock index at discharge as predictors of long-term mortality after myocardial infarction: results from the Augsburg Myocardial Infarction Registry

**DOI:** 10.1007/s00392-026-02929-z

**Published:** 2026-05-04

**Authors:** Constantin Rödl, Christa Meisinger, Bernhard Kuch, Philip Raake, Jakob Linseisen, Timo Schmitz

**Affiliations:** 1 Epidemiology, Faculty of Medicine, University of Augsburg, Stenglinstraße 2, 86156 Augsburg, Germany; 2 Department of Cardiology, Respiratory Medicine and Intensive Care, University Hospital Augsburg, Augsburg, Germany; 3 Stiftungskrankenhaus Nördlingen Department of Internal Medicine/Cardiology/Intensive Care, Donau-Ries-Kliniken, Nördlingen, Germany

**Keywords:** Myocardial infarction, Shock index, Modified shock index, Long-term mortality

## Abstract

**Background:**

Shock index (SI) and modified shock index (mSI), measured at hospital admission, have been shown to be predictive for mid- and long-term outcomes after acute myocardial infarction (AMI). Whether these associations also hold when indices are measured at discharge is unclear, so this study’s aim was to analyze the association between SI and mSI at discharge and long-term mortality after AMI.

**Material and methods:**

This analysis included 11,676 AMI cases registered by the population-based Myocardial Infarction Registry Augsburg. The follow-up time was restricted to a maximum of 5 years. Patients were categorized into low and high SI or mSI groups through separation at 75th percentiles for STEMI and NSTEMI, respectively. Analysis of survival included Kaplan–Meier curves with log-rank tests and multivariable-adjusted Cox-regression models.

**Results:**

Cut-off values were 0.6667 (STEMI) and 0.6545 (NSTEMI) for SI and 0.9231 (STEMI) and 0.9120 (NSTEMI) for mSI. Kaplan–Meier analysis showed significantly higher mortality for high SI and mSI groups in STEMI and NSTEMI patients. In STEMI cases, multivariable-adjusted Cox-regression analyses revealed significantly higher mortality for the high SI group (hazard ratio (HR): 1.25 (1.02–1.53), *p* value: 0.030), while mSI was non-significantly associated with long-term mortality (HR: 1.21 (0.99–1.48), *p* value: 0.060). Neither SI nor mSI was independently associated with mortality in NSTEMI.

**Conclusion:**

SI and mSI at discharge represent valuable tools for long-term post-infarction risk stratification especially in STEMI cases and can support decision-making regarding individualized ambulatory care.

**Graphical Abstract:**

**Figure:**
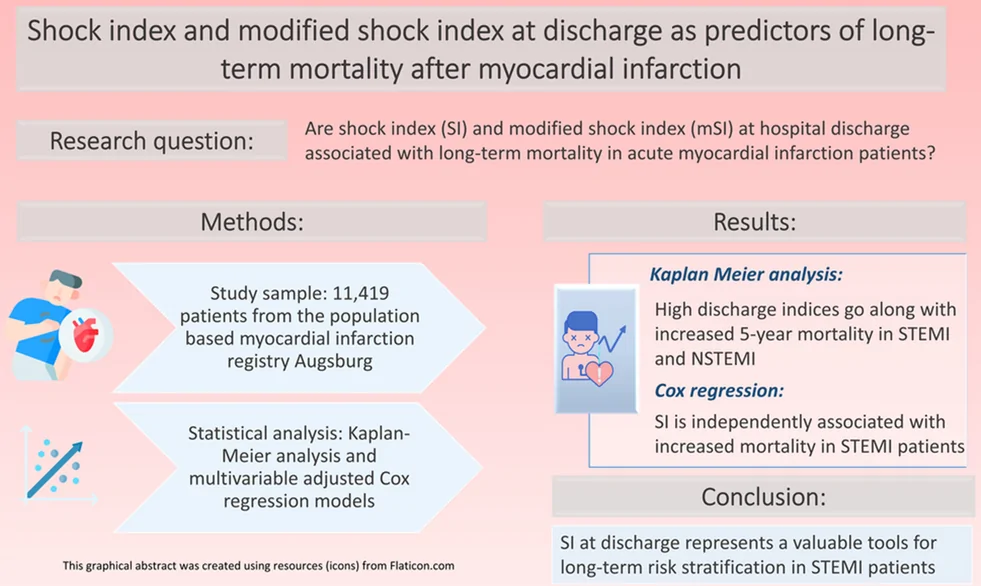

**Supplementary Information:**

The online version contains supplementary material available at https://doi.org/10.1007/s00392-026-02929-z.

## Introduction

Due to the persistent burden of acute myocardial infarction (AMI) for public health [1], practicable and clinically suitable tools for individual risk stratification remain relevant beyond the index hospitalization due to AMI. Heart rate and blood pressure are clinical parameters that reflect cardiovascular status and are part of basic clinical monitoring. These parameters can be combined in the shock index (SI) and modified shock index (mSI). The SI was developed already in 1967 by Allgöwer and Burri as heart rate divided by systolic blood pressure [2]. Liu et al. (2012) introduced the mSI, defined as the ratio of heart rate and mean arterial blood pressure (MAP), and thereby also considering diastolic blood pressure [3]. Originally developed as indices for emergency situations, both markers have been shown to be strong predictors for short-term mortality after AMI [2–4]. Reinstadler and coworkers demonstrated that SI is a predictor of major adverse cardiac events up to 1 year post-infarction [5]. Recently, our working group reported their predictive value for long-term mortality (up to several years) [6]. While recent studies analyzed SI and mSI separately for STEMI (ST-elevation myocardial infarction) and NSTEMI (non-ST-elevation myocardial infarction) [6], earlier research mostly did not differentiate between infarction types [7]. Furthermore, the primary research focus has been on SI and mSI at hospital admission, with limited evidence regarding their prognostic value at discharge. Since discharge reflects the patient’s status of cardiovascular recovery following therapy and marks the final point of in-hospital surveillance, evaluating the prognostic value of SI and mSI at this time is particularly relevant for planning ambulatory aftercare. Therefore, the aim of this study was to analyze whether SI and mSI at discharge can be used to stratify individual risks regarding long-term mortality after AMI, and whether their predictive strength differs between STEMI and NSTEMI, using data from a population-based registry with long-term follow-up.

## Materials and methods

### Patients

The research for this analysis was based on the Myocardial Infarction Registry Augsburg, which was established in 1984 as part of the MONICA project. The registry captures AMI cases in the city of Augsburg and the two surrounding counties (overall population of about 705,000). Inclusion required residency in the study region and age 25–74 (until 2008) or 25–84 years (from 2009 to 2017). Standardized bedside interviews by trained study nurses and systematic elaboration of the medical chart yielded detailed information on demographics, comorbidities, risk factors, diagnostics, and treatment. Information on vital status and dates of death was ascertained through data from residents’ registration and local health authorities. For this analysis, the latest comprehensive mortality update was performed in 2019. Further details on methodology and data collection can be found in previous publications [8, 9]. The registry protocol was approved by the Ethics Committee of the Bavarian Medical Association (BLÄK, ethics committee number: 12057, date of approval (latest amendment): 10. December 2024); all data was collected in accordance with the Declaration of Helsinki. The study was registered at the German Register of Clinical Studies (DRKS, project number DRKS00029042). Written consent was obtained from all patients.

The present analysis included all hospitalized patients with AMI between 2000 and 2017, who were discharged alive and survived at least 28 days after the AMI, thereby focusing exclusively on long-term mortality. Patients who died within the first 28 days were excluded. STEMI cases were defined as a physician-confirmed diagnosis of an acute coronary syndrome accompanied by new ST-segment elevations in the admission ECG (ST-segment elevations in 2 or more contiguous leads greater than 0.1 mV at the J point) and elevated cardiac enzymes; NSTEMI cases included all cases of acute coronary syndromes with elevated cardiac enzymes and no or unspecific ECG changes.

We excluded all patients with missing values for heart rate or systolic blood pressure at hospital discharge. The last documented values for resting blood pressure and heart rate on the day of discharge were used for calculation of SI and mSI. Different devices were used for the measurements, which were subject to regular calibration and quality check protocols. All measurements were obtained by trained nursing staff.

The SI was defined as heart rate divided by systolic blood pressure (SBP, in mmHg). For mSI, systolic blood pressure was replaced by mean arterial pressure (MAP), which further includes the diastolic blood pressure (DBP, in mmHg): MAP = (2 × DBP + SBP)/3.

We used the 75th percentile within each infarction type (STEMI/NSTEMI) as the cut-off value for dichotomization of patients, resulting in four distinct thresholds (SI-STEMI, SI-NSTEMI, mSI-STEMI, mSI-NSTEMI). Patients with index values at or below the 75th percentile were assigned to the low group, while those above the 75th percentile were assigned to the high group.

### Statistical analysis

Continuous variables were reported as median and inter-quartile range (IQR) due to non-normal distributions. Following a normal distribution, age was presented as mean and standard deviation. Categorical variables were shown as absolute frequencies and percentages. Group differences were tested using the Mann–Whitney *U* test for continuous variables and the *χ*^2^ test for categorical variables.

### Survival analysis

For all survival analyses, the follow-up duration was censored at 5 years. Unadjusted Kaplan–Meier curves were generated and stratified for low and high groups of SI and mSI. Log-rank test was used to test differences in long-term survival between both groups. This was done separately for the STEMI and the NSTEMI patients. Associations between SI or mSI and long-term mortality were further investigated using multivariable-adjusted Cox regression models for STEMI and NSTEMI cases separately. The following covariables were prespecified based on clinical relevance and prior literature to control for confounding while minimizing model complexity and overfitting: sex, age, diabetes, smoking status (current smoker, former smoker, never smoker, and unknown smoking status), hypertension, percutaneous coronary intervention (PCI), and beta blockers at discharge. The proportional hazards assumption for the covariables in all Cox-regression models was verified graphically using log–log-survival curves and by time-interaction terms in Cox regression models. Two-sided *p* values < 0.05 were considered statistically significant.

In a sensitivity analysis, the Cox models were calculated as described above but additionally adjusted for peak CK-MB levels (U/L).

All analyses were performed using IBM SPSS Statistics, version 30.0.0 and R version 4.4.3.

## Results

**Fig. 1:**
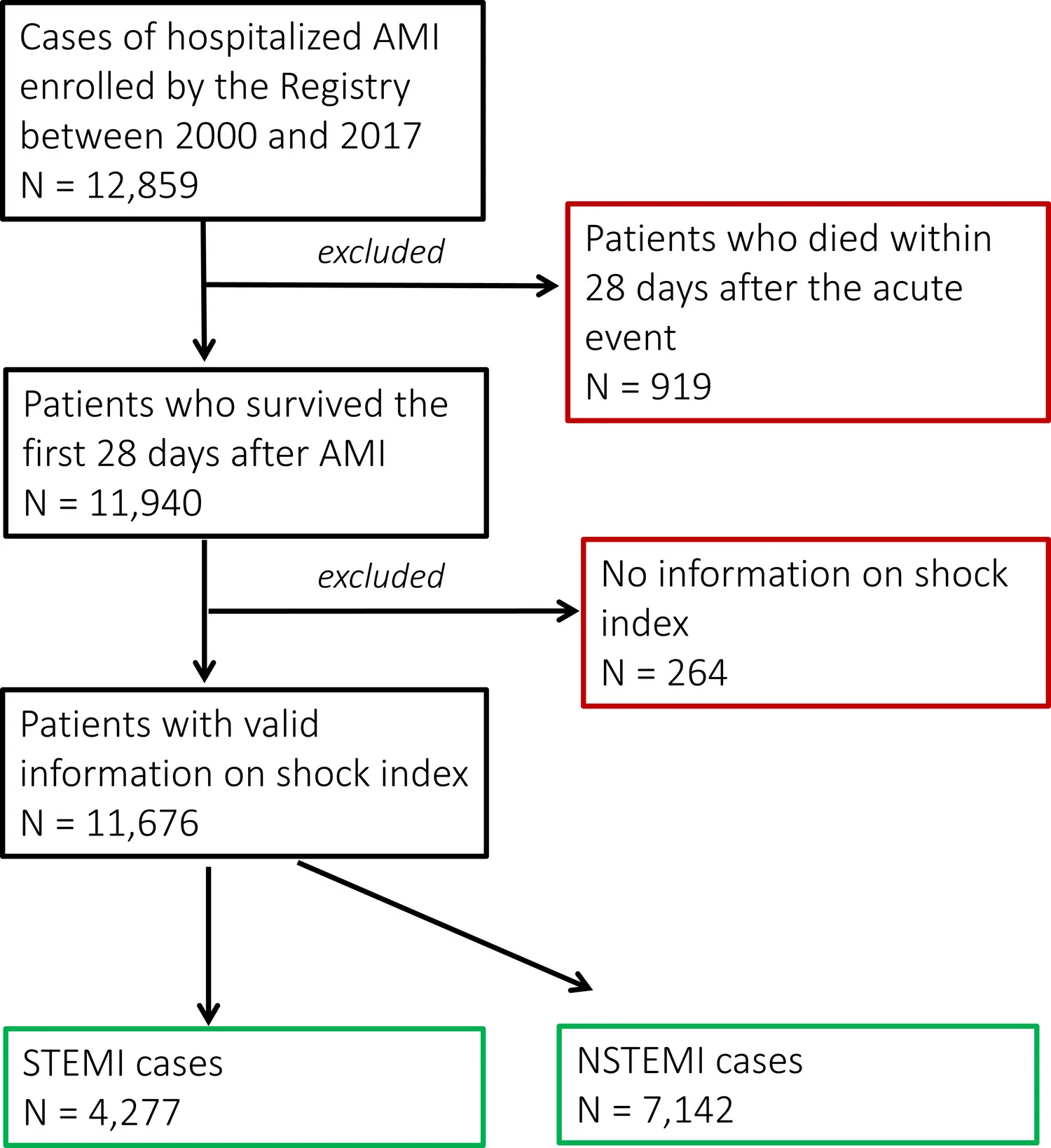
Flow chart of all inclusions/exclusions and the final study sample size

After excluding all patients who died within the first 28 days (919 out of 12,859), a total of 11,940 cases remained for the analysis of long-term survival, see Fig. 1. An additional 264 cases were excluded due to missing information on SI, resulting in 11,676 cases included in the statistical analysis (4277 STEMI cases, 7142 NSTEMI cases). The maximum follow-up time was 5.0 years (IQR: 2.9–5.0). The cut-off values used to stratify patients into high- and low-index groups were 0.6667 (STEMI) and 0.6545 (NSTEMI) for SI and 0.9231 (STEMI) and 0.9120 (NSTEMI) for mSI. Supplementary Table S1 shows the cross-tabulation of high and low groups for SI and mSI, illustrating that a substantial number of patients were classified as high for one index but low for the other index.

### Baseline characteristics

Baseline characteristics for the STEMI and NSTEMI patients, stratified by high/low SI groups, are displayed in Table 1. While patients in the high SI group were significantly younger in STEMI and NSTEMI cases, the proportion of women was significantly higher in the high SI group only in STEMI patients. Regarding comorbidities, patients with high SI had lower rates of diabetes and hypertension. Patients in the high index groups had higher discharge heart rates and lower blood pressure. Moreover, patients with high SI were less likely to be treated with PCI (STEMI: 77.4% vs. 83.9%; NSTEMI: 49.6% vs. 65.8%) and were slightly more often discharged with fewer medications, especially ATII antagonists and statins. Supplementary Table S2 provides baseline characteristics stratified by mSI groups.

**Table 1:** Baseline characteristics of patients, regarding SI and STEMI/NSTEMI cases given as frequencies (%), mean (SD), or median (IQR)

	STEMI				NSTEMI			
	Overall sample (*n* = 4277)	SI ≤ 0.6667 (*n* = 3271)	SI > 0.6667 (*n* = 1006)	*p* value	Overall sample (*n* = 7142)	SI ≤ 0.6545 (*n* = 5433)	SI > 0.6545 (*n* = 1709)	*p* value
Sex male (***n*** (IQR))	3150 (73.6)	2435 (74.4)	715 (71.1)	0.038	5114 (71.6)	3909 (71.9)	1205 (70.5)	0.262
Age (years; mean (SD))	61.6 (11.4)	62.1 (11.3)	60.1 (11.6)	< 0.001	65.2 (10.9)	65.6 (10.8)	63.7 (11.0)	< 0.001
Vital parameters								
Heart rate (bpm^a^; median (IQR))	72 (64–76)	68 (58–76)	80 (64.5–95.5)	< 0.001	72.0 (64–76)	68.0 (62–72)	80.0 (76–88)	< 0.001
Systolic blood pressure (mmHg; median (IQR))	120 (110–130)	120 (110–130)	105 (100–110)	< 0.001	120 (110–130)	120 (120–135)	110 (100–120)	< 0.001
Diastolic blood pressure (mmHg; median (IQR))	70 (60–80)	70 (60–80)	60 (60–70)	< 0.001	70 (60–80)	70 (65–80)	65 (60–70)	< 0.001
SI^b^ (median (IQR))	0.60 (0.53–0.67)	0.57 (0.51–0.62)	0.73 (0.70–0.80)	< 0.001	0.58 (0.51–0.65))	0.55 (0.50–0.60)	0.72 (0.68–0.77)	< 0.001
mSI^c^ (median (IQR))	0.82 (0.74–0.92)	0.78 (0.72–0.85)	1.01 (0.96–1.09)	< 0.001	0.81 (0.72–0.91)	0.77 (0.69–0.83)	0.99 (0.93–1.08)	< 0.001
Comorbidities								
Hypertension (***n*** (%))	3083 (72.1)	2448 (74.8)	635 (63.1)	< 0.001	5787 (81.0)	4511 (83.0)	1276 (74.7)	< 0.001
Diabetes (***n*** (%))	1183 (27.7)	934 (28.6)	249 (24.8)	0.018	2427 (34.0)	1884 (34.7)	543 (31.8)	0.030
Hyperlipidemia (***n*** (%))	2565 (60.0)	1980 (60.5)	585 (58.2)	0.178	4462 (62.5)	3424 (63.0)	1038 (60.7)	0.091
Smoking status (***n*** (%))								
Current smoker	1662 (38.9)	1226 (37.5)	436 (43.3)	0.003	1995 (27.9)	1420 (26.1)	575 (33.6)	< 0.001
Ex-smoker	1127 (26.4)	876 (26.8)	251 (25.0)	-	2301 (32.2)	1761 (32.4)	540 (31.6)	-
Never smoker	1250 (29.2)	992 (30.3)	258 (25.6)	-	2159 (30.2)	1747 (32.2)	412 (24.1)	-
No information on smoking status	238 (5.6)	177 (5.4)	61 (6.1)	-	687 (9.6)	505 (9.3)	182 (10.6)	-
Clinical characteristics								
Typical chest-pain symptoms^d^ (***n*** (%))	3744 (88.0)	2890 (88.8)	854 (85.4)	0.005	5627 (79.4)	4319 (80.1)	1308 (77.2)	0.013
Peak CK-MB levels (U/l; median (IQR))	110 (46–226)	106 (44.5–214)	123 (48.5–266.5)	< 0.001	35 (17–73)	34 (17–69)	38 (19–83)	< 0.001
Left ventricular ejection fraction (***n*** (%))				< 0.001				< 0.001
< 30%	232 (5.4)	140 (4.3)	92 (9.1)		333 (4.7)	199 (3.7)	134 (7.8)	
≥ 30%	3292 (77.0)	2556 (78.1)	736 (73.2)		5177 (72.5)	3997 (73.6)	1180 (69.0)	
No information	753 (17.6)	575 (17.6)	178 (17.7)		1632 (22.9)	1237 (22.8)	395 (23.1)	
Total days of hospital stay (median (IQR))	9.0 (7.0–14.0)	9.0 (7.0–13.0)	11.0 (8.0–17.0)	< 0.001	9.0 (7.0–15.0)	9.0 (6.0–14.0)	12.0 (8.0–20.0)	< 0.001
Total days in intensive care (median (IQR))	2.0 (1.0–3.0)	2.0 (1.0–3.0)	2.0 (1.0–4.0)	< 0.001	2.0 (1.0–4.0)	2.0 (1.0–3.0)	2.0 (1.0–5.0)	< 0.001
Treatment								
PCI^e^ (***n*** (%))	3521 (82.3)	2743 (83.9)	778 (77.4)	< 0.001	4420 (61.9)	3572 (65.8)	848 (49.6)	< 0.001
Bypass therapy (***n*** (%))	408 (9.5)	253 (7.7)	155 (15.4)	< 0.001	1243 (17.4)	681 (12.5)	562 (32.9)	< 0.001
I.V. thrombolysis therapy (***n*** (%))	126 (2.9)	94 (2.9)	32 (3.2)	0.691	29 (0.4)	21 (0.4)	8 (0.5)	0.807
Any reperfusion therapy (***n*** (%))	3953 (92.4)	3024 (92.4)	929 (92.3)	0.968	5608 (78.5)	4233 (77.9)	1375 (80.5)	0.028
Medication at discharge								
ACE blockers (***n*** (%))	3378 (80.4)	2607 (81.0)	771 (78.4)	0.082	5058 (73.0)	3885 (73.6)	1173 (70.8)	0.025
ATII antagonist (***n*** (%))	327 (7.8)	277 (8.6)	50 (5.1)	< 0.001	705 (10.2)	601 (11.4)	104 (6.3)	< 0.001
Beta blockers (***n*** (%))	4040 (96.1)	3106 (96.5)	934 (94.9)	0.029	6476 (93.4)	4943 (93.7)	1533 (92.5)	0.111
Antiplatelet drug (***n*** (%))	4127 (98.2)	3168 (98.4)	959 (97.5)	0.056	6687 (96.5)	5099 (96.6)	1588 (95.9)	0.183
Statins (***n*** (%))	3876 (92.3)	2999 (93.2)	877 (89.2)	< 0.001	6124 (88.3)	4711 (89.3)	1413 (85.4)	< 0.001

^a^Beats per minute

^b^Shock index

^c^Modified shock index

^d^Defined as chest pain or a feeling of pressure or tightness behind the breastbone

^e^Percutaneous coronary intervention

### Cox regression and Kaplan–Meier curves

**Fig. 2:**
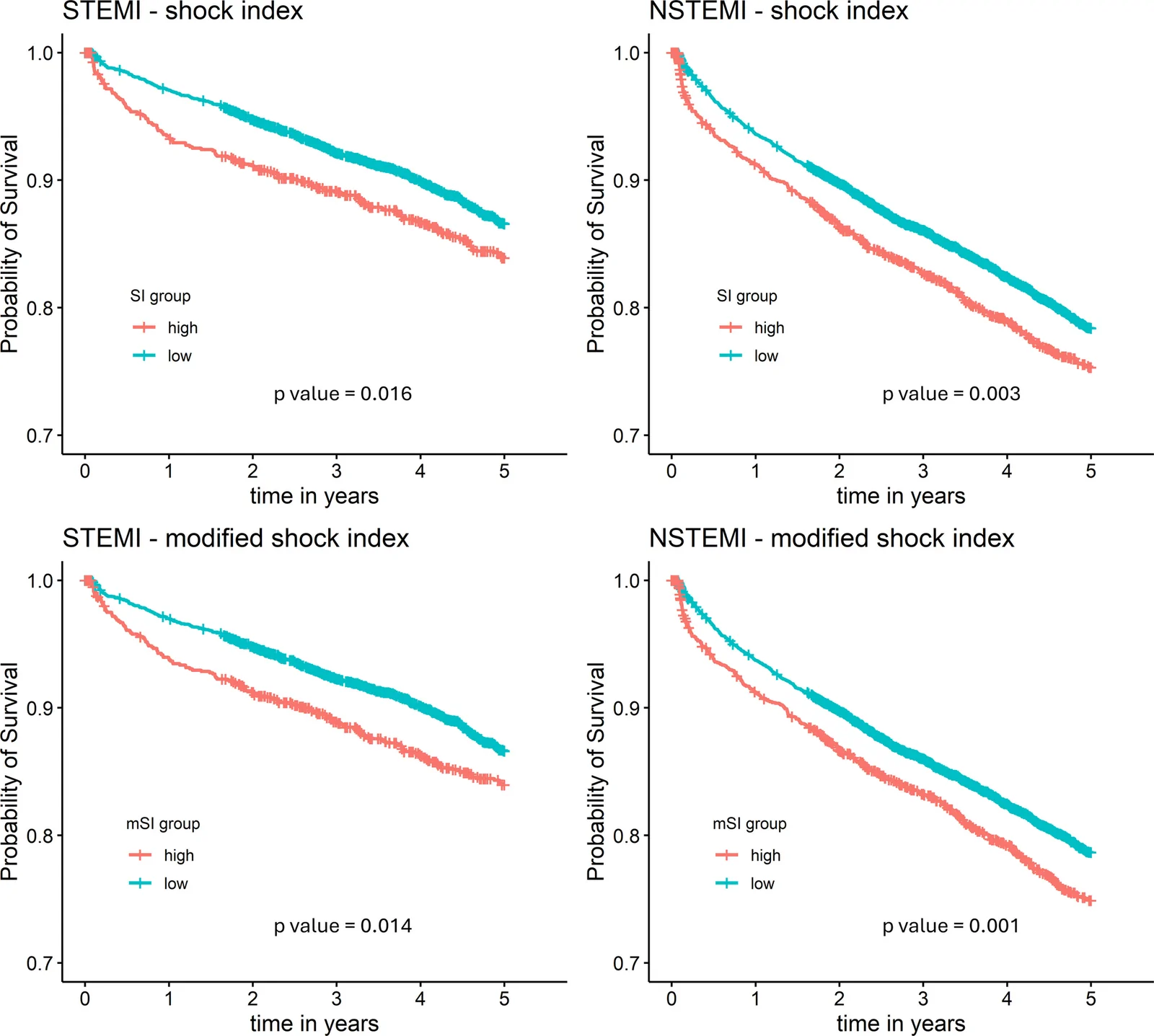
Kaplan–Meier survival curves stratified by low/high SI and mSI groups for STEMI and NSTEMI

Figure 2 shows Kaplan–Meier curves indicating significantly higher mortality in the groups with high indices. In all four groups, the survival curves diverged early and remained separated throughout the 5-year follow-up. Although there were slight differences between the plots, the degree of divergence was generally similar for both infarction types (NSTEMI, STEMI) and for both SI and mSI.

In multivariable Cox regression models, higher SI was significantly associated with an increased risk of 5-year mortality in STEMI patients (hazard ratio (HR): 1.25 (1.02–1.53), *p* value: 0.030), see Table 2. In STEMI patients, mSI was also associated with higher long-term mortality, but association did not reach statistical significance (HR: 1.21 (0.99–1.48), *p* value: 0.060). For NSTEMI cases, neither high SI nor high mSI were independently associated with increased long-term mortality. The full results of the SI models, including all confounders for STEMI and NSTEMI, are provided in Supplementary Table S3. Including peak CK-MB levels (U/l) during hospitalization as an additional confounder did not substantially change the effect estimates (see supplementary Table S4).

**Table 2:** Results of multivariable Cox regression models with 5-year all-cause mortality as end point. The models were adjusted for sex, age, diabetes, smoking status (current smoker, former smoker, never smoker and unknown smoking status), hypertension, percutaneous coronary intervention (PCI), and beta blockers at discharge

	High SI		High mSI	
	HR (95% CI)	*p*	HR (95% CI)	*p*
STEMI	1.25 (1.02–1.53)	0.030	1.21 (0.99–1.48)	0.060
NSTEMI	1.07 (0.95–1.22)	0.277	1.07 (0.94–1.21)	0.300

## Discussion

This study demonstrated that AMI patients with elevated SI and mSI values at discharge experience significantly higher long-term mortality in Kaplan–Meier analysis. The survival curves indicate that the greatest difference in mortality was in the first year after hospital discharge, but the curves remained separated for the entire 5-year follow-up time. The differences in mortality were smaller in NSTEMI cases compared to STEMI cases. For the latter, the association between SI and long-term mortality remained significant even after adjustment for major confounders, which was not the case for NSTEMI cases.

Most previous studies have found that admission SI and mSI are strong predictors for early outcomes and are useful in emergency triage situations after AMI [7]. Especially for STEMI cases, our analysis extends this knowledge to the point of discharge, which addresses a significant gap in the present scientific literature. Previous research focused heavily on admission values, leaving the discharge situation largely unexplored, not only in AMI but also across multiple cardiovascular pathologies. However, the observed results partially align with previous findings by Alapati et al., who found that higher discharge heart rates were independently associated with higher 3-year mortality after AMI [10].

Comparisons between high and low SI groups revealed notable differences in key characteristics, such as age, comorbidities, and discharge medication, which may partially explain the observed differences in long-term mortality in Kaplan–Meier analysis. For example, patients in the high SI groups were significantly less likely to receive statins at discharge—a difference not directly attributable to blood pressure or risk of hypotension—suggesting that there are important underlying group differences that may have influenced the observed outcomes.

Apart from that, several physiological mechanisms may underlie the results seen in this study. The indices measured at discharge likely reflect a combination of post-intervention cardiovascular stress, medication effects, volume status, and subclinical dysfunctions that persisted throughout hospitalization [11, 12]. Clinical evidence indicates that AMI often causes chronically increased sympathetic and reduced parasympathetic activity, which can result in persistently elevated heart rates and the occurrence of arrhythmias and progressive heart failure [13–15]. Other studies have shown that persistent tachycardia, a driving factor for increased SI and mSI, is associated with pathological cardiac remodeling and an increased myocardial oxygen demand [16, 17]. Therefore, higher SI and mSI values during hospitalization and especially at discharge may reflect ongoing cardiovascular stress or dysfunction, which could be associated with increased mortality. Importantly, these considerations remain hypothetical, given that our observational analysis did not include measurements of biomarkers, autonomic nervous system activity, or myocardial remodeling.

The present analysis showed that SI and mSI were more predictive in STEMI patients than in NSTEMI patients. In a previous analysis based on data from the Augsburg Myocardial Infarction Registry, we reported that SI and mSI, measured at hospital admission, were significantly associated with long-term mortality in multivariable Cox regression models with a slightly better predictive value in NSTEMI cases compared to STEMI cases, which is the other way round compared to what we found in the present analysis [6]. The current findings might be explained through greater infarction severity and transmural ischemia in STEMI patients [18]. Contrarily, NSTEMI patients present a highly heterogenous group with higher comorbidity burdens and more complex cardiac profiles [19]. In NSTEMI cases, admission SI and mSI seem to be much more predictive compared to the corresponding discharge indices. For STEMI cases, discharge indices might provide (additional) predictive information regarding long-term mortality.

### Clinical implications

As previously mentioned, SI and mSI are resource-efficient tools, as they are derived from routine vital parameters collected during standard clinical monitoring. Especially in STEMI cases, these indices might be valuable measures for long-term mortality, specifically in the first year after the event (the Kaplan–Meier curves suggest the greatest effect in the early phase of the 5-year follow-up period). Integration of these indicators into discharge check lists could potentially support risk stratification and help to determine post-discharge care intensity and follow-up patterns in cooperation with primary care providers. Automated calculation of the indices within electronic health records systems would efficiently position them as additional information for assessing discharge readiness, complementing rather than replacing existing tools. Although scores like GRACE and TIMI remain more relevant in the present context of prognostic risk stratification, the emergence of artificial intelligence in medical contexts including AI-based early warning systems [20] opens new opportunities for incorporating additional indices. Including SI and mSI at discharge alongside other known predictors could enhance clinical decision-making without adding diagnostic or administrative complexity. The indicated implications suggest that SI and mSI could be implemented across different healthcare infrastructures. In resource-limited settings, such as small hospitals, even manual calculation from routinely obtained clinical parameters is an option. Conversely, hospitals with advanced IT infrastructures can implement automated calculations into decision-support systems.

### Further research implications

Further research is necessary to externally validate the present findings. Investigating the dynamics of SI and mSI throughout the clinical stay as well as combining these indices with laboratory markers and ECG-derived features could provide additional insights into their predictive value for long-term mortality. Since SI and mSI measured at hospital admission as well as hospital discharge are associated with mortality after AMI and especially after STEMI, the analysis of whether the predictive strength of both indices could be transferred to the rehabilitation process and throughout later points of aftercare would be of interest. Additionally, experimental or imaging studies could help bridge the gap between the observed indices and underlying pathophysiological processes.

### Strengths and limitations

The strengths of this analysis primarily result from qualities of the Augsburg Myocardial Infarction Registry, which features a real-world setting, a large cohort, and long follow-up periods with consecutive and standardized data collection protocols. The population-based approach of the registry minimizes selection bias, ensuring a representative sample which carefully suggests transferability to other healthcare systems and aftercare structures. Furthermore, the extensive information collected for each patient enabled the adjustment for relevant confounders.

However, several limitations must be considered. First, the outcome in the present analysis was all-cause mortality, without differentiation between cardiac and non-cardiac causes. Second, changes in medication and therapeutic interventions during the follow-up period, especially the introduction of new medication classes, were not considered. Third, despite adjustment in the Cox regression models, residual and unmeasured confounding cannot be ruled out, particularly regarding factors such as ethnicity, socioeconomic status, or post-discharge medical care structures. In particular, there was no information on medication adherence, participation in rehabilitation programs, or lifestyle modification after discharge, all of which could have affected the relationship between the discharge SI/mSI and long-term mortality. Also, the models were not adjusted for measures of heart failure (e.g., left-ventricular ejection fraction), which may be relevant confounders. Moreover, no information on (high-sensitivity) cardiac troponin was available for this analysis. Finally, although all data were collected by medically trained staff, measurement errors or variations in the timing of measurements in relation to discharge could have influenced the accuracy and comparability of SI and mSI.

## Conclusion

The present findings showed that in both STEMI and NSTEMI cases, high SI and mSI at discharge are associated with significantly higher long-term mortality in Kaplan–Meier analysis. Additionally, SI at discharge is an independent predictor of long-term all-cause mortality in STEMI patients. As directly implementable hemodynamic metrics derived from standard vital parameters, discharge indices may be useful for individualized risk stratification especially after STEMI, particularly when combined with other prognostic factors.

## Supplementary Information

Below is the link to the electronic supplementary material.

ESM 1 Supplementary Material 1 (DOCX 116 KB)

## Data Availability

The datasets generated during and/or analyzed during the current study are not publicly available.
