# Supplementary material for: Shock index and modified shock index at discharge as predictors of long-term mortality after myocardial infarction: results from the Augsburg Myocardial Infarction Registry
Source: Clin Res Cardiol. 2026 May 4;115(11):1973–82. doi: 10.1007/s00392-026-02929-z (PMC13658313; doi:10.1007/s00392-026-02929-z)
Supplement: Supplementary file 1 [file 392_2026_2929_MOESM1_ESM.docx]

**Supplements**

**Title: Shock index and modified shock index at discharge as predictors of long-term mortality after myocardial infarction: results from the Augsburg Myocardial Infarction Registry**

Constantin Rödl, Christa Meisinger, Bernhard Kuch, Philip Raake, Jakob Linseisen, Timo Schmitz

**Table S1:** Classification of patients into SI and mSI groups stratified for STEMI/NSTEMI

| STEMI | | |  |
| --- | --- | --- | --- |
|  | high SI | low SI |  |
| high mSI | 858 | 204 | 1062 |
| low mSI | 146 | 3066 | 3212 |
|  | 1004 | 3270 | Total: 4274 |
| NSTEMI | | |  |
|  | high SI | low SI |  |
| high mSI | 1396 | 373 | 1769 |
| low mSI | 308 | 5059 | 5367 |
|  | 1704 | 5432 | Total: 7136 |

**Table S2:** Baseline characteristics of patients stratified for mSI group and STEMI/ NSTEMI

|  | ***STEMI*** | | | ***NSTEMI*** | | |
| --- | --- | --- | --- | --- | --- | --- |
|  | ***mSI ≤ 0.9231 (n=3271)*** | ***mSI > 0.9231 (n=1006)*** | ***p-value*** | ***mSI ≤ 0.9120 (n=5367)*** | ***mSI > 0.9120 (n=1769)*** | ***p-value*** |
| ***sex male (n (IQR))*** | *2394 (74.5)* | *753 (70.9)* | *0.022* | *3849 (71.7)* | *1261 (71.3)* | *0.749* |
| ***age (years; mean (SD))*** | *62.0 (11.3)* | *60.4 (11.7)* | *<0.001* | *65.5 (10.8)* | *64.1 (11.0)* | *<0.001* |
| ***Vital parameters*** | | | | | | |
| ***Heart rate (bpm^1^; median (IQR))*** | *68 (62 - 72)* | *80 (72 - 86)* | *<0.001* | *68 (60- 72)* | *80 (74 - 86)* | *<0.001* |
| ***Systolic blood pressure (mmHg; median (IQR))*** | *120 (110 - 130)* | *110 (100 - 120)* | *<0.001* | *120 (120 - 135)* | *110 (100 - 120)* | *<0.001* |
| ***Diastolic blood pressure (mmHg; median (IQR))*** | *70 (65 - 80)* | *60 (60 - 70)* | *<0.001* | *70 (70 - 80)* | *60 (60 - 70)* | *<0.001* |
| ***SI^2^ (median (IQR))*** | *0.57 (0.51 - 0.62)* | *0.73 (0.68 - 0.80)* | *<0.001* | *0.55 (0.49 - 0.60)* | *0.71 (0.67 - 0.77)* | *< 0.001* |
| ***mSI^3^ (median (IQR))*** | *0.78 (0.71 - 0.84)* | *1.01 (0.96 - 1.09)* | *<0.001* | *0.77 (0.69 - 0.83)* | *0.99 (0.95 - 1.07)* | *< 0.001* |
| ***Comorbidities*** | | | | | | |
| ***hypertension (n (%))*** | *2414 (75.2)* | *666 (62.7)* | *<0.001* | *4455 (83.0)* | *1327 (75.0)* | *<0.001* |
| ***diabetes (n (%))*** | *917 (28.5)* | *265 (25.0)* | *0.026* | *1853 (34.5)* | *572 (32.4)* | *0.100* |
| ***hyperlipidemia (n (%))*** | *1953 (60.8)* | *610 (57.4)* | *0.057* | *3408 (63.5)* | *1051 (59.4)* | *0.002* |
| ***Smoking status (n (%))*** | | | | | | |
| *current smoker* | *1198 (37.3)* | *463 (43.6)* | *<0.001* | *1415 (26.4)* | *579 (32.7)* | *< 0.001* |
| *ex-smoker* | *867 (27.0)* | *260 (24.5)* | *-* | *1725 (32.1)* | *576 (32.6)* | *-* |
| *never smoker* | *979 (30.5)* | *269 (25.3)* | *-* | *1716 (32.0)* | *439 (24.8)* | *-* |
| *no information on smoking status* | *168 (5.2)* | *70 (6.6)* | *-* | *511 (9.5)* | *175 (9.9)* | *-* |
| ***Clinical characteristics*** | | | | | | |
| ***typical chest-pain symptoms^4^ (n (%))*** | *2839 (88.7)* | *902 (85.7)* | *0.009* | *4266 (80.1)* | *1358 (77.4)* | *0.020* |
| ***Peak CK-MB levels (U/l; median (IQR))*** | *106 (45 - 214)* | *123 (47 - 258)* | *0.001* | *34 (17 - 69)* | *39 (18 - 85)* | *<0.001* |
| ***Left ventricular ejection fraction (n (%))*** |  |  | *<0.001* |  |  | *<0.001* |
| ***< 30%*** | *131 (4.1)* | *100 (9.4)* |  | *203 (3.8)* | *130 (7.3)* |  |
| ***≥ 30%*** | *2518 (78.4)* | *773 (72.8)* |  | *3946 (73.5)* | *1226 (69.3)* |  |
| ***No information*** | *563 (17.5)* | *189 (17.8)* |  | *1218 (22.7)* | *413 (23.3)* |  |
| ***Total days of hospital stay (median (IQR))*** | *9.0 (7.0 - 13.0)* | *11.0 (8.0 - 17.0)* | *<0.001* | *9.0 (6.0 - 14.0)* | *12.0 (8.0 - 20.0)* | *<0.001* |
| ***Total days in intensive care (median (IQR))*** | *2.0 (1.0 - 3.0)* | *2.0 (1.0 - 4.0)* | *<0.001* | *2.0 (1.0 - 3.0)* | *2.0 (1.0 - 5.0)* | *<0.001* |
| ***Treatment*** | | | | | | |
| ***PCI^5^ (n (%))*** | *2697 (84.0)* | *821 (77.4)* | *<0.001* | *3554 (66.3)* | *865 (48.9)* | *<0.001* |
| ***Bypass therapy (n (%))*** | *236 (7.3)* | *172 (16.2)* | *<0.001* | *658 (12.3)* | *581 (32.8)* | *<0.001* |
| ***i.v. thrombolysis therapy (n (%))*** | *90 (2.8)* | *36 (3.4)* | *0.380* | *21 (0.4)* | *8 (0.5)* | *0.893* |
| ***Any reperfusion therapy (n (%))*** | *2963 (92.2)* | *987 (92.9)* | *0.503* | *4188 (78.0)* | *1415 (80.0)* | *0.088* |
| ***Medication at discharge*** | | | | | | |
| ***ACE blockers (n (%))*** | *2564 (81.1)* | *812 (78.4)* | *0.065* | *3849 (73.8)* | *1209 (70.4)* | *0.006* |
| ***ATII antagonist (n (%))*** | *275 (8.7)* | *52 (5.0)* | *<0.001* | *603 (11.6)* | *102 (5.9)* | *<0.001* |
| ***beta blockers (n (%))*** | *3049 (96.4)* | *989 (95.4)* | *0.163* | *4888 (93.7)* | *1586 (92.3)* | *0.044* |
| ***antiplatelet drug (n (%))*** | *3118 (98.6)* | *1007 (97.1)* | *0.003* | *5037 (96.6)* | *1649 (96.0)* | *0.305* |
| ***statins (n (%))*** | *2955 (93.4)* | *919 (88.7)* | *<0.001* | *4658 (89.3)* | *1465 (85.4)* | *<0.001* |

^1^ beats per minute

^2^ shock index

^3^ modified shock index

^4^ defined as chest pain or a feeling of pressure or tightness behind the breastbone

^5^ percutaneous coronary intervention

**Table S3:** Total results of multivariable Cox regression models for STEMI and NSTEMI analyzing the association between SI group and long-term mortality.

|  | **STEMI** | | **NSTEMI** | |
| --- | --- | --- | --- | --- |
|  | HR (95% CI) | p | HR (95% CI) | p |
| SI group high | 1.25 (1.02-1.53) | 0.030 | 1.07 (0.95-1.22) | 0.277 |
| age | 1.09 (1.07-1.10) | < 0.001 | 1.09 (1.08-1.10) | < 0.001 |
| sex (female) | 1.06 (0.87-1.29) | 0.565 | 0.94 (0.83-1.07) | 0.336 |
| diabetes | 1.37 (1.14-1.65) | < 0.001 | 1.71 (1.53-1.91) | < 0.001 |
| hypertension | 1.03 (0.82-1.29) | 0.808 | 0.87 (0.74-1.02) | 0.080 |
| current smoker | 1.50 (1.16-1.94) | 0.002 | 1.82 (1.52-2.18) | < 0.001 |
| ex smoker | 1.38 (1.09-1.75) | 0.008 | 1.47 (1.27-1.70) | < 0.001 |
| no information on smoking | 3.40 (2.51-4.61) | < 0.001 | 2.85 (2.39-3.38) | < 0.001 |
| PCI | 0.60 (0.49-0.74) | < 0.001 | 0.55 (0.49-0.61) | < 0.001 |
| beta blockers at discharge | 0.76 (0.53-1.09) | 0.134 | 0.75 (0.62-0.91) | 0.003 |

**Table S4:** Results of multivariable Cox regression models with five-year all-cause mortality as end point. The models were adjusted for sex, age, diabetes, smoking status (current smoker, former smoker, never smoker and unknown smoking status), hypertension and percutaneous coronary intervention (PCI), beta blockers at discharge and peak CK-MB levels (U/l).

|  | **High SI** | | **High mSI** | |
| --- | --- | --- | --- | --- |
|  | HR (95% CI) | p value | HR (95% CI) | p value |
| STEMI | 1.22 (0.99 – 1.50) | 0.059 | 1.20 (0.96 – 1.47) | 0.086 |
| NSTEMI | 1.05 (0.92 - 1.22) | 0.430 | 1.05 (0.92 - 1.21) | 0.469 |
